# Polypharmacy in African American Adults: A National Epidemiological Study

**DOI:** 10.3390/pharmacy7020033

**Published:** 2019-03-29

**Authors:** Shervin Assari, Hamid Helmi, Mohsen Bazargan

**Affiliations:** 1 Department of Family Medicine, Charles R. Drew University of Medicine and Sciences, Los Angeles, CA 90095, USA; mohsenbazargan@cdrewu.edu; 2 Center for Research on Ethnicity, Culture and Health, School of Public Health, University of Michigan, Ann Arbor, MI 48109-2029, USA; 3 School of Medicine, Wayne State University, Detroit, MI 48202, USA; hhelmi@umich.edu; 4 Department of Family Medicine, University of California Los Angeles, Los Angeles, CA 90095, USA

**Keywords:** ethnicity, race, African Americans, Blacks, medications, medication use, polypharmacy, healthcare use

## Abstract

**Background:** Despite the association between polypharmacy and undesired health outcomes being well established, very little is known about epidemiology of polypharmacy in the African American community. We are not aware of any nationally representative studies that have described the socioeconomic, behavioral, and health determinants of polypharmacy among African Americans. **Aims:** We aimed to investigate the socioeconomic and health correlates of polypharmacy in a national sample of African American adults in the US. **Methods:** The National Survey of American Life (NSAL, 2003–2004) included 3,570 African American adults. Gender, age, socioeconomic status (SES; education attainment, poverty index, and marital status), access to the healthcare system (health insurance and having a usual source of care), and health (self-rated health [SRH], chronic medical disease, and psychiatric disorders) in addition to polypharmacy (5 + medications) as well as hyper-polypharmacy (10 + medications) were measured. Logistic regressions were applied for statistical analysis. **Results:** that About 9% and 1% of all African American adults had polypharmacy and hyper-polypharmacy, respectively. Overall, higher age, higher SES (education and poverty index), and worse health (poor SRH, more chronic medical disease, and psychiatric disorders) were associated with polypharmacy and hyper-polypharmacy. Individuals with insurance and those with a routine place for healthcare also had higher odds of polypharmacy and hyper-polypharmacy. **Conclusions:** Given the health risks associated with polypharmacy, there is a need for systemic evaluation of medication use in older African Americans with multiple chronic conditions. Such policies may prevent medication errors and harmful drug interactions, however, they require effective strategies that are tailored to African Americans.

## 1. Background

Despite the use of various definitions across studies [1], polypharmacy reflects a high risk of inappropriate use of medications [2,3]. Commonly defined as concomitant use of several medicines [4], polypharmacy is associated with several health threats including drug-drug interactions and reduced adherence to essential medications [5,6]. Polypharmacy is also linked to cognitive decline and unintentional falls [5,6], which are major risk factors for morbidity and mortality of older adults [7]. Polypharmacy also imposes additional economic burden on the patients, individuals, and the healthcare system, as well as on society [8]. As a result, it is essential to conduct national epidemiological studies to generate accurate knowledge on the prevalence and correlates of polypharmacy in the community [9].

Polypharmacy may increase the risks of hospitalization [10] and mortality [10,11] in older adults. These effects are at least in part because polypharmacy increases adverse drug reactions in older individuals by 75% [12,13], which is important for the following two inter-related reasons. First, drug interactions are responsible for a major proportion of hospital admissions of older adults, and second, almost half of such hospitalizations are preventable, if we can prevent inappropriate drug interactions [14]. 

Polypharmacy is particularly prevalent in older adults [15,16,17,18], as they receive multiple prescribed medications [16] for their diagnosed chronic medical [1,19,20,21] and psychiatric [22] conditions. The prevalence of polypharmacy is also several times higher in the presence of multi-morbidities and comorbidities [17,18]. In many industrial countries, as the population ages, the prevalence of polypharmacy is rising too [23].

In addition to a high prevalence, older people are also more vulnerable to the adverse effects of polypharmacy due to their fragility [24]. Age-related physiological changes make older adults prone to medical complications of polypharmacy, as compared to younger individuals [24]. Race/ethnic minorities, people with low socioeconomic status (SES), and individuals with low health literacy, and cognitive dysfunction are particularly prone to adverse events due to polypharmacy [24].

For a number of reasons, polypharmacy is a major threat to African American individuals [25]. First, African Americans are less likely to receive new, most effective, and simplified medication combinations [26,27,28,29,30]. A lack of access to such combined/simple medication regimens likely results in their use of regimens composed of multiple, older, complex, and generic medications [4,25]. Such complexity of dosing regimens may increase their risk of inappropriate medication use and decrease their medication adherence [31]. From the pool of epidemiological studies that have focused on polypharmacy [32,33,34], very few are conducted on African Americans [4,25]. Among this limited pool of studies, we are not aware of any study with a nationally representative sample.

Research on risk factors of polypharmacy and hyper-polypharmacy in the African American community helps us identify subgroups of individuals who are at an increased risk of inappropriate medication use [35,36,37]. Such knowledge can inform local and national strategies that aim to eliminate racial disparities that exist in polypharmacy among older adults in the U.S. The results derived from such studies may be useful to address the unmet needs of the African American community, through first designing and then implementing and evaluating evidence-based clinical, community based, and public health programs and interventions that can specifically reduce potentially inappropriate medication use in high risk African Americans. Unfortunately, very little information exists on subgroups of African Americans have an increased risk of polypharmacy [4,25,38,39].

As mentioned earlier, only a small number of epidemiological studies have ever examined polypharmacy in African American adults [25,38,39]. From this pool, most studies have recruited a local sample, leaving a remaining need to conduct studies in nationally representative samples. On a sample of older African Americans in South Los Angeles (*n* = 400), Bazargan et al., [25] showed that 75% and 30% of all the participants were taking 5+ (polypharmacy) and 10+ (hyper-polypharmacy) medications per day, which is above the national rates [25]. The same study estimated that about 7 of 10 participants may have inappropriate drug use. In that study, gender, number of healthcare providers, comorbid conditions, and potentially inappropriate use of medications were correlated with polypharmacy in African American older adults [25]. There is still a need to study research on the epidemiology of polypharmacy in African American adults.

### Aims

Using a nationally representative sample, we aimed to investigate the demographic, socioeconomic, healthcare access, and health determinants of polypharmacy (5 + medications) and hyper-polypharmacy (10 + medications) among African American adults.

## 2. Materials and Methods

### 2.1. Design and Setting

The National Survey of American Life (NSAL 2003) [40,41,42] is a large national health survey of African American adults in the US. As a part of the Collaborative Psychiatric Epidemiology Surveys (CPES 2003), NSAL was supported by the National Institute of Mental Health (NIMH) [40,41,42]. 

### 2.2. Ethics

The Institutional Review Board (IRB) of the University of Michigan (UM), Ann Arbor, approved the NSAL study protocol (# B03-00004038-R1). Participants provided informed written consent. All the individuals received a financial incentive for their time. Data were collected, restored, and analyzed in a confidential manner. 

### 2.3. Participants and Sampling

The NSAL included a total number of 3570 African American adults. All participants were at least 18 years old. African Americans were drawn from large cities, other urban areas, and also rural areas. The NSAL sampling was a national household probability sample of White and Blacks/African Americans. Inclusion criteria were English speaker, non-institutionalized, resident of the U.S., and being able and willing to consent. 

### 2.4. Data Collection

Approximately 82% of all the interviews were face-to-face which were performed in participants’ homes. The 18% remaining interviews were conducted by phone. NSAL applied Computer Assisted Personal Interviews (CAPIs) to facilitate the process of interview and enhance the data quality. The CAPI is one of the preferred methods of interviewing when the survey tool is lengthy and complex [43]. All the interviews were conducted in English language. Interviews took 100 min on average to complete. The response rate was about 71% for African Americans.

### 2.5. Race and Ethnicity

Race/ethnicity of the participants in the NSAL was self-identified. African Americans were Blacks who did not have ancestral ties to any Caribbean countries.

### 2.6. Survey Measures and Study Variables

#### 2.6.1. Demographic Characteristics

Demographic characteristics measured for this study were age and gender. Age was treated as an interval variable, while gender was a dichotomous variable (1 male, 0 female).

#### 2.6.2. Socioeconomic Status

Socioeconomic status indicators in this study included educational attainment and poverty status (income to needs ratio). Income to needs ratio, calculated based on number of the individuals in the household and household income was an interval measure, with a higher score indicating higher SES. The study defined years of schooling as a measure of SES, which was treated as an interval variable.

#### 2.6.3. Access to the Healthcare System

Two indicators of access were considered: insurance status and usual place of care. Insurance status and usual place of care were both conceptualized as dichotomous variables. 

#### 2.6.4. Self-Rated Health (SRH)

Participants were asked to rate their health. Responses were “excellent, very good, good, fair, or poor”. The exact item read as “How would you rate your overall physical health at the present time? Would you say it is excellent, very good, good, fair or poor?” SRH can be operationalized as a dichotomous or numerical value [44,45,46], we treated SRH as a nominal variable. Fin our study, we compared “poor health” to any other levels (i.e., excellent, very good, good, or fair). This cutoff point is commonly used in the literature and is shown to predict mortality and health [47,48,49,50,51,52,53].

#### 2.6.5. Chronic Medical Disease (CMC)

In this study we measured the prevalence of CMC by measuring self-reported history of doctor-diagnosed history of the following 14 chronic diseases: “diabetes, arthritis/rheumatism, peptic ulcers, cancer, hypertension, chronic liver disease, chronic kidney disease, stroke, asthma, other chronic lung diseases, atherosclerosis, sickle cell disease, heart disease, and glaucoma”. Participants were asked “whether a doctor has ever told them that they have the above listed conditions”. We calculated a sum score for CMC, with a potential range of 0 to 14, with a high score indicating a higher number of CMCs present. In this study, CMC was treated as an interval variable [54,55,56,57,58,59,60,61,62,63,64]. Self-reported measures of CMC are believed to be valid and reliable [64].

#### 2.6.6. Psychiatric Disorders

To evaluate the endorsement of criteria for lifetime psychiatric disorders, the World Mental Health Composite International Diagnostic Interview (WMH-CIDI) was used. CIDI is based on the Diagnostic and Statistical Manual of Mental Disorders, Fourth Edition (DSM-IV). The following psychiatric disorders were assessed: “mood disorders (major depressive disorder, dysthymia, and bipolar disorders); anxiety disorders (generalized anxiety disorder, posttraumatic stress disorder, panic disorder, agoraphobia, social phobia, and obsessive compulsive disorder); substance use disorders (alcohol abuse/dependence and drug abuse/dependence); eating disorders (anorexia nervosa, bulimia nervosa, binge-eating disorder) and childhood disorders (oppositional defiant disorder, conduct disorder, separation anxiety disorder, and attention deficit/hyperactivity disorder)” [65,66,67,68,69,70,71]. This is a common way of treating psychiatric disorders in the literature [72,73].

#### 2.6.7. Polypharmacy and Hyper-Polypharmacy

Polypharmacy was defined as taking 5+ medications [74]. Hyper-polypharmacy was defined as taking 10+ medications [74]. Participants were asked to report their medication use over the past seven days: “How many different kinds of prescription medicine have you taken during the past seven days?” The interviewees were instructed that prescription medicines are those that can be only obtained from doctors and requires doctors’ written approval or prescriptions to a pharmacist. Participants were also asked to report any medicine even if it was used only once.

### 2.7. Statistical Analysis

To adjust for the NSAL complex survey design, we used Stata 15.0 (Stata Corp., College Station, TX, US) for data analysis. Taylor series technique was used for re-estimation of the NSAL design-based standard errors (SEs). All the percentages, means, associations, and p values are nationally representative. 

For our multivariable analyses, we applied survey logistic regressions, using sub-pop commands. In our models, polypharmacy or hyper-polypharmacy were the main dependent variables, demographic factors (age and gender), SES (educational attainment, poverty status, and marital status), access to the healthcare system (health insurance status and having a usual place of care), and health (number of chronic diseases, psychiatric disorders, and SRH) were the independent variables. Odds ratio (OR), standard errors (SE), and p values were reported. A *p*-value equal or smaller than 0.05 were considered as significant.

## 3. Results

### 3.1. Descriptive Statistics

The sample included 3570 African American adults. Table 1 describes distribution of demographic factors, SES indicators, healthcare access, health status, polypharmacy, and hyper-polypharmacy in the pooled sample and by gender.

Table 1 suggests that 9.32% and 1.05% of African Americans report polypharmacy and hyper-polypharmacy, respectively. These prevalence rates were 10.74% and 1.40% for African American women and 7.51% and 0.60% for African American men.

African American men were more likely to be married and reported higher income to needs ratio (*p* < 0.05). African American women were more likely to report a usual source of care compared to African American men (*p* < 0.05). African American women reported more CMC than African American men (*p* < 0.05). Polypharmacy or hyper-polypharmacy were also more common in African American women than in African American men (*p* < 0.05).

### 3.2. Determinants of Polypharmacy in the Pooled Sample

Table 2 presents the results of logistic regression models with polypharmacy as the outcome in the pooled sample and by gender. Based on *Model 1* which was in the pooled sample, age, access to the healthcare system (health insurance status and having a usual place for healthcare), and health (SRH, more chronic medical disease, and psychiatric disorders) were associated with polypharmacy. Older individuals, those with insurance, those with a routine place for healthcare, those who perceived their health as poor, individuals with a history of psychiatric disorder, and individuals who had more chronic medical conditions were more likely to report polypharmacy. Gender and SES (education and poverty index) were not associated with polypharmacy in the sample. (Table 2)

### 3.3. Determinants of Polypharmacy in Males and Females

Table 2 also shows the results of logistic regressions with polypharmacy as the outcome in males and females. In females, age, insurance, routine place of care, and all health indicators were correlated with polypharmacy. In males, however, only age and chronic medical conditions but not healthcare access, SRH, or lifetime psychiatric disorders were associated with polypharmacy. (Table 2)

### 3.4. Determinants of Hyper-Polypharmacy in the Pooled Sample

Table 3 shows the summary of the results of two logistic regression models with hyper-polypharmacy as the outcome in the pooled sample as well as by gender. Based on *Model 1* which was in the pooled sample, age, gender SES (education attainment), healthcare access (usual place of care), and health (SRH and chronic medical disease) were associated with hyper-polypharmacy. Older individuals, females, those with a routine place for healthcare, those who perceived their health as poor, and individuals who had more chronic medical conditions had higher odds of hyper-polypharmacy. Insurance, poverty status, and lifetime psychiatric disorders were not associated with hyper-polypharmacy in the sample. (Table 3)

### 3.5. Determinants of Hyper-Polypharmacy in Males and Females

Table 3 also shows the results of two logistic regression models with hyper-polypharmacy as the outcome in males and females. In females, health indicators were all correlated with hyper-polypharmacy. In males, SRH and chronic medical disease but not lifetime psychiatric disorders were associated with hyper-polypharmacy. In neither males nor females, age, SES, and healthcare access factors were correlated with hyper-polypharmacy. (Table 3)

## 4. Discussion

We used a national sample to investigate how demographic, socioeconomic, healthcare access, and health factors shape social patterning of polypharmacy and hyper-polypharmacy among African American adults. The study also explored gender differences in this regard. 

Findings suggest that age, gender, SES (education attainment), healthcare access (health insurance and having a usual place of care), and health (SRH, chronic medical disease, and psychiatric disorders) were associated with polypharmacy and hyper-polypharmacy. However, these associations differed between polypharmacy and hyper-polypharmacy as well as by gender. For example, being female was associated with hyper-polypharmacy but not with polypharmacy. Among main observed gender differences, we found that endorsing criteria for a psychiatric disorder (lifetime) was associated with polypharmacy and hyper-polypharmacy in women but not men. Similarly, women, but not men, who perceived their health to be poor were at a higher risk of polypharmacy. Among factors that were universally associated with polypharmacy and hyper-polypharmacy was number of chronic medical diseases, showing association regardless of gender and polypharmacy level.

Among demographic factors that correlate with polypharmacy is gender [75,76]. Although not all research agrees on this issue [77], some studies have documented a higher prevalence of polypharmacy for women than men [75,76,77,78]. Women are more commonly exposed to almost all types of inappropriate drug use [75,76]. Some studies have suggested that the association between gender and potentially inappropriate drug use is above and beyond confounders such as age, SES, and chronic disease [76]. Women better follow the prescriptions of doctors [79]. In one study, elderly women with low education were at a higher risk of polypharmacy, hyper-polypharmacy, and potential inappropriate drug use than men with low education [80]. In another study, men were at a higher risk of cardiac polypharmacy, however, women had a higher risk of non-cardiac polypharmacy [23]. Thus, most of the literature suggests that women are at a relative disadvantage compared to males when it comes to potentially inappropriate drug use. 

Compared to men, women report more chronic disease [81] Women also more commonly seek healthcare for their conditions [82]. Women are also better aware of their symptoms [83] and better communicate with doctors about symptoms [84]. For males, however, healthcare utilization may be perceived as weakness [85,86,87], thus men have fewer diagnosed conditions than women [88]. Even when diagnosed, men show worse adherence to treatments [89]. Men also tend to delay healthcare use [90]. Women are particularly prone to be given multiple/inappropriate psychotropic drugs [75]. These differences are partially because women more easily adopt the sick role; so they recognize and accept their experiences that reflect a health problem. These are socially learned behaviors due to gendered socialization [91,92].

Age [76] is another factor commonly linked to polypharmacy. Although some of the effect of age on polypharmacy is because of number of chronic diseases, the link between age and polypharmacy in our study remained significant above and beyond chronic diseases. As individuals age, chronic diseases accumulate over time; such growing catalogue of conditions mean more pharmacological treatments prescribed for the individual [19]. As people reach their early sixties, the majority of individuals have at least two diagnosed chronic disorders that require treatment [20]; and as individuals reach their late sixties, more than 25% of the populations have three or more diagnosed chronic disease that require treatment [21]. Age of development of chronic disease is several years younger in African Americans compared to Whites [93], suggesting that screening for polypharmacy may be needed for middle age African Americans. 

We found that higher rather than lower education is a risk factor for hyper-polypharmacy in African Americans, a link that could not be observed for polypharmacy. Among 621 older adults (aged > or = 77 years) who were selected from Swedish Panel Study of Living Conditions of the Oldest Old, a nationally representative sample of Swedish older adults, low education was a risk factor for polypharmacy. However, this association disappeared after controlling for confounders such as physical health [94]. In another study, high level of education reduced the likelihood of polypharmacy, independent of disease burden [23]. It is unclear why we found a positive rather than a negative link between education and hyper-polypharmacy in African Americans. One hypothesis is that education enhances African American population’s access to healthcare, so their medical conditions are diagnosed and treated more frequently. 

One of the factors linked to polypharmacy in the current study was regular place of care. Although older African American individuals are less likely than White individuals to have a “regular care provider”, having a “regular place of care” may increase their risk for inappropriate use of prescribed medications [95]. Among African American older adults in Los Angeles, Bazargan showed documented number of healthcare providers as the strongest determinant of polypharmacy [25]. Thus, healthcare access influence polypharmacy, even in African Americans who are at a relative disadvantage compared to Whites regarding healthcare access. 

The results may help with the design of interventions that may reduce polypharmacy in African Americans [96,97]. Bazargan showed that the prevalence of polypharmacy is higher in African American older adults compared to national norms (NHANES) [25]. A meta-analysis that systemically reviewed 25 studies on reduction of polypharmacy in older adults found that the existing programs and interventions are not efficient in reducing unnecessary polypharmacy [98], suggesting that there is a need for future research on ways that may help us achieve a reduction of inappropriate polypharmacy [98]. Thus, the next step of research is to conduct interventional studies that may discover practical solutions to reduce polypharmacy [25].

Although polypharmacy is an increasing [99] cause of morbidity and mortality among the elderly [99], we still do not have a detailed understanding of epidemiology of this problem in African Americans. We also do not have culturally acceptable evidence-based programs that can reduce inappropriate drug use in this population.

### 4.1. Implications

The findings reported here have some public health implications that can contribute to the elimination of health disparities in African Americans. To find potentially inappropriate drug use, we need to conduct a comprehensive evaluation of medications among African Americans who are older, have high SES, have multiple medical and psychiatric conditions, and have a usual source of care.

### 4.2. Limitations

The current study was not without limitations. First of all, the design of this study was cross-sectional. Thus, we cannot infer any causal associations. Future research is needed using longitudinal design. Second, our outcome was based on self-reports of number of medications. There is a need to assess medications and not rely on patients’ self-reported data. Third, many relevant confounders such as health literacy and number of healthcare providers were not used in this study. We also did not fully investigate availability and usage of healthcare services, pathways to service, and type of medical and psychiatric conditions. Data should be collected on type of medication, and inappropriate medication use. The variables were also limited to individual level, we did not collect contextual and provider characteristics. Future research should investigate all potential factors that can potentially impact inappropriate drug use. The study used old data that was collected over 15 years ago. The outdated data may cause unnecessary confusion, thus there is a need for following analysis. The readers should be cautious with interpretation of the findings as some differences are likely to have happened over the past 15 years. Research using data from recent years may be compared with the results reported here to understand whether major changes in the correlates of polypharmacy has happened over 15 years. Despite the above limitations, the results of this study extend the existing literature on epidemiology and correlates of polypharmacy in African Americans [25,100]. 

Using crude data on polypharmacy might provide conflicting information for healthcare providers. Such data should be added by more nuances on inappropriate polypharmacy. Thus, the results should be regarded as preliminary. Still, our findings have the potential to serve as a foundation for other studies, for example, looking at insurance claims to corroborate medication adherence. Various sources of bias and unmeasured confounding suggest that the interpretation of the findings should be associated with caution. Although the results and conclusions are still valid, it should be noted that using various types of data collection on polypharmacy may result in conflicting information. This is another reason the results should be interpreted with caution to avoid any confusion for healthcare providers.

### 4.3. Future Research

This study only measured polypharmacy, not unnecessary medication use or harmful, unnecessary polypharmacy. Thus, future research should be conducted on inappropriate medication use. In addition, we are aware of the fact that various medical conditions differ regarding their need for medications. The type of chronic medications may also differ across races. That means, differences in types of chronic disease may result in differences between races in polypharmacy and also correlates of polypharmacy. Thus, future research should investigate the types of conditions as well. A more comprehensive analysis is needed and may provide more precise knowledge on the links between race, SES, health, and polypharmacy. It would be important to compare ethnic groups to determine if there are health inequalities leading to polypharmacy or indeed under prescribing.

## 5. Conclusions

In summary, although with some gender differences, demographic, SES, healthcare access, and health factors are associated with polypharmacy among African Americans. Given the health risks associated with polypharmacy, there is a need to comprehensively evaluate medication use to prevent medication errors and harmful drug interactions in African Americans. Such an aim requires culturally acceptable and effective interventions that can prevent inappropriate medication use and drug interactions among African Americans.

## Figures and Tables

**Table 1:** Descriptive results in the overall sample (*n* = 3570).

	African Americans (*n* = 3570)		*Women* (*n* = 2299)		*Men* (*n* = 1271)	
	***% (SE)***	***95% CI***	***% (SE)***	***95% CI***	***% (SE)***	***95% CI***
Gender						
Female	44.03 (0.01)	42.35–45.73				
Male	55.97 (0.01)	54.27–57.65				
Any Psychiatric Disorders						
No	60.65 (0.01)	58.28–62.98	59.74 (0.01)	57.34–62.09	61.82 (0.02)	57.35–66.10
Yes	39.35 (0.01)	37.02–41.72	40.26 (0.01)	37.91–42.66	38.18 (0.02)	33.90–42.65
Married *						
No	58.22 (0.01)	56.07–60.34	64.52 (0.01)	61.83–67.11	50.22 (0.02)	46.86–53.57
Yes	41.78 (0.01)	39.66–43.93	35.48 (0.01)	32.89–38.17	49.78 (0.02)	46.43–53.14
Routine Source of Care *						
No	13.95 (0.01)	12.22–15.88	9.72 (0.01)	8.24–11.44	19.32 (0.01)	16.47–22.53
Yes	86.05 (0.01)	84.12–87.78	90.28 (0.01)	88.56–91.76	80.68 (0.01)	77.47–83.53
Insurance						
No	18.14 (0.01)	16.37–20.05	17.74 (0.01)	15.22–20.57	18.64 (0.01)	16.69–20.76
Yes	81.86 (0.01)	79.95–83.63	82.26 (0.01)	79.43–84.78	81.36 (0.01)	79.24–83.31
Polypharmacy *						
No	90.68 (0.01)	89.59–91.68	89.26 (0.01)	87.64–90.69	92.49 (0.01)	90.51–94.09
Yes	9.32 (0.01)	8.32–10.41	10.74 (0.01)	9.31–12.36	7.51 (0.01)	5.91–9.49
Hyper-Polypharmacy *						
No	98.95 (0.01)	98.23–99.39	98.60 (0.00)	97.73–99.14	99.40 (0.00)	98.69–99.73
Yes	1.05 (0.01)	0.61–1.77	1.40 (0.00)	0.86–2.27	0.60 (0.00)	0.27–1.31
	***Mean (SE)***	***95% CI***	***Mean***	***95% CI***	***Mean***	***95% CI***
Age *	42.04 (0.53)	40.96–43.13	42.27 (0.57)	41.11–43.42	41.75 (0.67)	40.39–43.12
Education Attainment *	12.46 (0.08)	12.29–12.63	12.46 (0.10)	12.26–12.66	12.46 (0.12)	12.23–12.70
Poverty Index (High SES)	2.65 (0.09)	2.46–2.84	2.30 (0.09)	2.12–2.47	3.10 (0.13)	2.84–3.35
Number of CMC	1.26 (0.03)	1.21–1.31	1.40 (0.03)	1.34–1.47	1.07 (0.04)	0.99–1.16

Source: The National Survey of American Life (NSAL, 2003–2004), CMC: Chronic Medical Conditions, SES: Socioeconomic Status, CI: Confidence Interval, OR: Odds Ratio, * *p* < 0.05.

**Table 2:** Factors associated with polypharmacy in the pooled sample and by gender.

	All		Females		Males	
	OR(SE)	p	OR(SE)	p	OR(SE)	p
Gender (Female)	1.25(0.25)	0.290	-	-	-	-
Age	1.03(0.01)	<0.001	1.02(0.01)	<0.001	1.03(0.01)	0.006
Education Attainment	1.13(0.10)	0.180	1.10(0.14)	0.441	1.18(0.14)	0.160
Poverty Status (High SES)	1.05(0.03)	0.124	1.02(0.04)	0.666	1.07(0.05)	0.132
Marital Status (Married)	0.92(0.17)	0.666	1.08(0.25)	0.751	0.71(0.15)	0.124
Insurance (Any)	1.99(0.58)	0.025	3.05(0.82)	<0.001	1.00(0.48)	0.992
Routine Place of Healthcare	2.86(1.16)	0.014	3.94(2.14)	0.017	2.27(1.36)	0.183
Self-Rated Health (Poor)	2.05(0.55)	0.012	2.40(0.64)	0.002	1.56(1.05)	0.511
Chronic Medical Conditions (n)	1.80(0.11)	<0.001	1.77(0.12)	<0.001	1.92(0.15)	0.000
Lifetime Psychiatric Disorders	1.45(0.25)	0.042	1.49(0.30)	0.050	1.37(0.39)	0.273
Intercept	0.00(0.00)	<0.001	0.00(0.00)	<0.001	0.00(0.00)	<0.001

Source: The National Survey of American Life (NSAL, 2003–2004), SES: Socioeconomic Status, SE: Standard Error, OR: Odds Ratio.

**Table 3:** Factors associated with hyper-polypharmacy in the pooled sample and by gender.

	All		Females		Males	
	OR(SE)	p	OR(SE)	p	OR(SE)	p
Gender (Female)	3.10(1.06)	0.002	-	-	-	-
Age	1.01(0.01)	0.297	1.02(0.01)	0.195	1.00(0.03)	0.890
Education Attainment	1.53(0.33)	0.050	1.38(0.25)	0.091	2.98(1.94)	0.101
Poverty Status (High SES)	0.95(0.07)	0.538	1.00(0.09)	0.960	0.72(0.27)	0.378
Marital Status (Married)	2.02(1.07)	0.192	1.59(1.12)	0.518	6.12(6.98)	0.121
Insurance (Any)	3.87(4.06)	0.206	3.66(3.81)	0.223	-	-
Routine Place of Healthcare	9.71(11.55)	0.064	6.21(7.28)	0.128	-	-
Self-Rated Health (Poor)	5.82(3.10)	0.002	3.57(1.89)	0.022	46.38(56.20)	0.003
Chronic Medical Conditions (n)	1.81(0.14)	<0.001	1.73(0.15)	<0.001	2.10(0.41)	0.001
Lifetime Psychiatric Disorders	1.82(0.69)	0.123	2.28(0.93)	0.050	0.45(0.47)	0.446
Intercept	0.00(0.00)	<0.001	0.00(0.00)	<0.001	0.00(0.00)	<0.001

Source: The National Survey of American Life (NSAL, 2003–2004), SES: Socioeconomic Status, SE: Standard Error, OR: Odds Ratio.
